# At risk but not adequately included: People with disabilities’ experience of COVID-19 in Zambia

**DOI:** 10.4102/ajod.v13i0.1448

**Published:** 2024-11-15

**Authors:** Queen E. Seketi, J. Anitha Menon, Charles Michelo, Lena Morgon Banks, Virginia Bond

**Affiliations:** 1 Department of Epidemiology and Biostatistics, School of Public Health, University of Zambia, Lusaka, Zambia; 2 Department of Psychology, School of Humanities and Social Sciences, University of Zambia, Lusaka, Zambia; 3 School of Liberal Studies, University of Petroleum and Energy Sciences, New Delhi, India; 4 Global Health Institute, Nkwazi Research University, Lusaka, Zambia; 5 School of Health Sciences, Chreso University, Lusaka, Zambia; 6 International Centre for Evidence in Disability, London School of Hygiene and Tropical Medicine, London, United Kingdom; 7 Social Science Unit, Zambart, Lusaka, Zambia; 8 Department of Global Health and Development, Faculty of Public Health and Policy, London School of Hygiene & Tropical Medicine, London, United Kingdom

**Keywords:** disability, COVID-19, experiences, knowledge, communication, risk, health seeking behaviour, social determinants, disability inclusion, health inequities

## Abstract

**Background:**

COVID-19 had an impact on all sections of society, including people with disabilities.

**Objectives:**

The authors aimed to explore the needs and experiences of people with disabilities in Zambia during the COVID-19 pandemic.

**Method:**

In this hermeneutic phenomenological study, we used a semi-structured interview guide to collect data from a purposive and snowball sample of 40 people with disabilities and their caregivers. The participants were from 11 districts in 6 provinces in Zambia. The in-depth interviews were done between July 2022 and November 2022. Data were managed in NVivo and analysed using reflexive thematic analysis.

**Results:**

The three themes included: (1) awareness and experience of public health measures on COVID-19 among people with disabilities; (2) experience of othering and stigmatisation as people with disability during the COVID-19 pandemic and (3) experience of COVID-19 symptoms and having COVID-19 among people with disabilities.

**Conclusion:**

Interventions were largely unresponsive to the needs of people with disabilities, exacerbating the risk of exposure to infection. In future, adaptations like emergency risk communication in braille, audio and sign language interpretation in adapted communication formats should be made. Further studies are needed to quantify the gaps in access to health, explore policies and strategies to improve health outcomes for people with disabilities in LMICs like Zambia.

**Contribution:**

The findings may contribute to the development and enhancement of policies and interventions responsive to the needs of people with disabilities in future pandemics in the Zambian context.

## Introduction

COVID-19 affected everyone (Finn & Andrew 2020), including people with disabilities. It is a respiratory infection that was declared as a pandemic by the World Health Organization (WHO) in March 2020 (Chipimo et al. 2020). At its height, the pandemic was the leading cause of morbidity and mortality in the adult population worldwide (Fan et al. 2021). People with disabilities are among the largest group of people marginalised during the COVID-19 pandemic (McKinney, McKinney & Swartz 2021), and there was compelling evidence on their increased morbidity and mortality in developed countries like the United Kingdom (Bosworth et al. 2021) and the United States of America (Landes et al. 2020). On the international development agenda, exclusion of people with disabilities is a major concern because of its association with multidimensional poverty which limits participation in mainstream life areas (Mitra, Posarac & Vick 2013). Countries are mandated to have disability-inclusive measures to address development concerns like inequities in access to health, education attainment, employment population ratio and food security (Mitra et al. 2023).

For example, the Zambian government, in collaboration with partners, implements social protection programmes that strive to target people with disabilities among other marginalised populations (MCDSS 2018). Additionally, Zambia’s legislative measures aim to domesticate the United Nations Convention on the Rights of Persons with Disability (UNCRPD). In its preamble, the UNCRPD states that disability is an evolving concept and that disability results from the interaction between people with impairments and attitudinal and environmental barriers, which hinders their full and effective participation in society on an equal basis with others (Lawson 2006). While the WHO’s International Classification of Functioning, Disability and Health (ICF) refers to disability as an umbrella term for impairments, activity limitations and participation restrictions. It denotes the negative aspects of the interaction between an individual with a health condition and that individual’s contextual factors (personal and environmental). Globally, at least 1.3 billion individuals or 16% of the population are people with disabilities (World Health Organization [WHO] 2021). In Zambia, about 1.5 million people or 7.7% of the population are people with disabilities (MCDSS 2018). Consequently, it is important to place greater emphasis on ensuring the inclusion of people with disabilities in various areas and development activities (Zambia Agency for Persons with Disabilities 2021).

The first reported case of COVID-19 in Zambia was in March 2020, with the first fatality following the same year (Chipimo et al. 2020). The WHO declared COVID-19 as a public health emergency on 11 March 2020 (WHO 2020); in turn, Zambia declared COVID-19 as a national emergency as of 19 March 2020. The Ministry of Health initiated COVID-19 responses immediately (Haider et al. 2020). A variety of public health and social measures were implemented in Zambia to stop the spread of COVID-19. These interventions included test, trace and treat, movement restrictions, and partial or complete closure of schools (Ministry of General Education Zambia 2020) and businesses. Additionally, quarantine measures were enforced in specific districts in the first wave, and international travel restrictions were put in place. The restrictions were varied as the pandemic unfolded (Chipimo et al. 2020). Communication on the public health crisis was made through public and private media platforms, social media and Ministry of Health messages to mobile numbers (Risk Communication and Community Engagement [RCCE] Subcommittee 2020). Guidelines focussed on ‘the five golden rules’, which were important behavioural interventions that individuals could follow, and included frequent handwashing with soap and water or the use of an alcohol-based hand sanitizer, keeping a safe distance from other people, using face masks, coughing in elbows, seeking early medical assistance and later (from April 2021 onwards) receiving the COVID-19 vaccine. By August 2023, there have been 349 287 reported cases of COVID-19 and 4069 deaths in Zambia (Mathieu et al. 2020).

However, people with disabilities could face additional barriers to following preventative measures, which may increase their risk of infection, and an even higher risk of serious illness and mortality if they receive suboptimal care and have underlying conditions (WHO 2020). For example, a study from the United States showed that 22% of people with disabilities found it difficult to obtain information about COVID-19 due in part to the lack of adapted communication (James et al. 2022), and a scoping review of 30 studies in low- and middle-income countries (LMICs) found reduced access to general healthcare (Rohwerder et al. 2022). Although people with disabilities were at risk of COVID-19 because of their narrower margin of health (Shakespeare, Ndagire & Seketi 2021), there are also underlying systematic barriers to accessing healthcare in general (Pincock et al. 2022), such as physical accessibility, communication barriers (Rohwerder et al. 2022), infrastructure accessibility, medical equipment, caregiver support and stigma and discrimination (Hashemi et al. 2022; Neille & Penn 2015). In crisis settings, the inequities faced by people with disabilities are exacerbated because of their exclusion from preparedness, response and recovery measures (World Bank Group & GFDRR 2017).

This article provides evidence for how COVID-19 public health prevention measures were experienced among people with disabilities in Zambia. In-depth interviews were used to explore their knowledge of COVID-19 and experiences following preventive measures in people with disabilities and/or their caregivers in 5 selected provinces (11 districts) of Zambia. It may carry implications for how pandemics can deepen inequities and how pandemic preparedness strategies should be adapted to be inclusive of people with disability.

## Methods

### Study design

We used a hermeneutic phenomenological study (González-Díaz et al. 2021) to explore experiences of people with disability and the biopsychosocial theory underpinning the ICF (WHO 2001). These were used in this qualitative study because we wanted to understand the lived experiences of people with disabilities during the COVID-19 pandemic, and the role of different personal and contextual factors in shaping this experience, from their own perspectives. Interviews used semi-structured, in-depth interview guides.

### Study setting, site and population

This study was undertaken during the different COVID-19 waves in 11 urban and rural districts across five provinces in Zambia. These included Lusaka, Chongwe, Kafue (Lusaka province); Mkushi, Mumbwa, Kabwe (Central province); Mongu (Western Province); Ndola and Chingola (Copperbelt province); Livingstone and Monze (Southern Province). The study population, included in this article, were people with disabilities and their caregivers.

### Participant selection

We used purposive sampling with maximum variation (Patton 2015) to select 40 people with different types of disability, aged 7 years and above (7 is the age at which children are either in reception class or grade 1 in Zambia); gender; level of support needed for daily living and geographical spread. These were drawn from lists provided by Organisations of People with Disabilities and Non-governmental Organisations focussed on disability registered with Zambia Agency for Persons with Disabilities. Proxies were persons directly involved in the care of young children (7–11 years) and people living with severe forms of impairments affecting communication; persons constantly present in the day-to-day lives of participants like parents and guardians and significantly involved in managing the person’s COVID-19 risk. All efforts were made to speak to the person living with a disability and three proxy interviews were the last resort. Refer to Table 1 in the findings section for details on characteristics of participants.

**TABLE 1:** Social demographic characteristics of people with disabilities (*N* = 40).

Variable	Frequency (*n*)	Proportion (%)
**Gender**		
Female	22	55.0
Male	18	45.0
**Religion**		
Christian	39	98.0
Muslim	1	2.0
**Disability type**		
Hearing impairment	5	12.5
Speech impairment	5	12.5
Vision impairment	9	22.5
Physical impairment	12	30.0
Developmental impairment or cerebral palsy	6	15.0
Intellectual impairment	3	7.5
**Area of residence**		
Urban	7	17.5
Peri-urban	18	45.0
Rural	15	37.5

Note: Participants’ sociodemographic characteristics, with age mean of 36 years; age range: 10–74 years.

### Data collection

Data collection was done through in-depth interviews between July 2022 and November 2022. Earlier, Q.E.S. (a female PhD student) received research training in qualitative research and disability-inclusive research practices from the University of Ghana by LMB (secondary supervisor) and a team of global health and disability researchers from the London School of Hygiene and Tropical Medicine (LSHTM) to sharpen her skills to conduct this study. Interviews were conducted in English, Nyanja, Tonga and sign language. The interview guide was pilot-tested, and the findings of that test were used to refine the tool. All interviews were audio-recorded and transcribed. The interview transcripts were kept on a password-protected computer in a safe place. Interviews were anonymised by the removal of personal identification features like names and were replaced with in-depth interview numbers. The data were checked for accuracy and consistency by Q.E.S. and the research assistants. L.M.B. and V.B. (supervisors) also conducted quality checks of the research. We interviewed a maximum of eight participants per impairment category hearing, (speech, vision, physical developmental and intellectual impairment) to cater for data saturation. These were recruited until no new relevant knowledge was forthcoming. See Table 1 for the profile of participants. The face-to-face interviews were done at the preferred, quiet venue of participants, for example, homes, at their office.

### Data analysis

We employed reflexive thematic analysis, following Braun and Clark’s six-phase approach for coding and theme development (Braun & Clarke 2006), which aligns with the reflexive requirements of phenomenological analysis.

### Ethical considerations

Regarding authority to conduct research, ethical clearance was obtained from the London School of Hygiene and Tropical Medicine (reference: 22616) and the University of Zambia Biomedical Research Ethics Committee, clearance number 1269-2020 and 3324-2022. Informed consent to participate was obtained directly from all participants above the age of 18 years in Zambia. For children below the age of 18 years and for adults who lacked the capacity to consent on their own (e.g. severe cognitive impairments), caregivers provided consent. In these cases, attempts were made to still seek input from participants directly where possible and relevant as in the cases of children aged 10 years and above. Proxy interviews were still needed for younger children and adults who had severe difficulties communicating with available support. Using simplified information sheets, assent from the participants aged 10–18 years was sought, following the same protocols for consent such as oral or emailed for phone interviews and written if the interview was conducted in person.

Adaptations were put in place to support the participation of people with different impairments. For example, people with profound hearing impairment could participate using sign language interpretation and through written responses over email and/or WhatsApp. Simplified interview schedules were used for people with cognitive and/or intellectual impairments and younger children. Job Access with Speech (JAWs), a screen reader, was installed on Q.E.S.’s computer. This was available to read information sheets and consent forms by participants with visual impairments if they wished. Personal assistants, although nonparticipants, were also allowed during interview. Some participants with visual impairments opted to read information sheets from their own smartphones and laptops. Referral services were identified and available if participants reported severe distress.

### Team reflexivity

A female PhD student, Q.E.S., led this study and has a background in development studies and public health, with more than 16 years of experience in the coordination of multisectoral response to human immunodeficiency virus (HIV) at the district level. In this study, Q.E.S. took an emic position. Ontologically, the emic account refers to an insider’s perspective (Markee 2013). This is because Q.E.S. is a Zambian woman with a physical disability who lived through the COVID-19 pandemic in Lusaka. Through face-to-face meetings, Q.E.S. and V.B. reflected on what went well, what did not go well and how we could do it better. Q.E.S.’s own assumptions of things being out there waiting to be discovered changed to a realisation that knowledge is a co-creation with participants, needing patience and engagement in cycles of reading, writing and re-writing. In this study, team members were focussed on a continuous effort to co-create and contribute to increased knowledge on disability inclusion during crises in the global south. Hence, a multidisciplinary team brought different expertise – for instance, VB is a qualitative expert who has worked in Zambia for 30+ years and LMB is a UK-based researcher who has 10+ years of experience in disability research globally. CM is a clinician and public health specialist and AJM is a social scientist with over 20 years of experience.

The research team had no prior relationship with participants. Q.E.S. took time to share the research objectives and ensure that they knew about the interviewer. C.K. (Public Health Undergraduate), M.M. (experienced in qualitative research) and V.K. (Public Relations Undergraduate) helped to set up interviews with participants and recordings. When Q.E.S. travelled out of the district, she was accompanied by a caregiver whose role was to help her navigate inaccessible physical environment and was not part of the interview session.

## Results

### Characteristics of the sample

Participants were nearly equally divided by gender and ranged in age from 10 to 74 years (mean age being 36 years; Table 1). Most (45%; 18/40) participants resided in peri-urban areas, 37.5% (15/40) in rural locations, and a few (17.5%; 7/40) in urban areas. Additionally, 98% (39/40) were Christians, while 2% (1/40) were Muslims. People with physical impairments were the largest share by disability type, although there was good representation across types.

To frame the analysis, it is important to note that most participants were living in difficult socioeconomic situations. Most participants reported that their households often had to make tough economic choices between buying the basics (e.g. food, utilities) and purchasing airtime, internet bundles and television subscriptions to stay abreast of COVID-19; this task became difficult during the pandemic. While peri-urban and urban residents had electricity connections, they experienced load shedding and high electricity tariffs. In rural areas, access to adequate water and sanitation was limited. Most participants were not receiving social welfare benefits from government because the criteria were to include people with ‘severe’ impairments, and this tends to leave out people with mild and moderate disabilities who may need welfare support. However, many received material support from religious organisations and organisations of people with disabilities. Most of the adult participants were unemployed or in informal work either in self-employment or part of cooperatives.

## Themes

This section presents findings on the in-depth interviews conducted with people with disabilities. Table 2 summarises the themes and subthemes.

**TABLE 2:** Key themes and subthemes.

Key themes	Subthemes
1. Awareness and Experience of Public Health Measures on COVID-19 among People with disabilities	1.1 Awareness of COVID-19 and accompanying public health measures among People with disabilities 1.2 Challenges of access to COVID-19 information 1.3 Practicalities of following the five golden rules on COVID-19 prevention
2. Experience of Othering and Stigmatisation as people with disability during the COVID-19 pandemic	2.1 Disability, COVID-19 stigmatisation 2.2 Being labelled as ‘the one spreading COVID-19’ 2.3 Neglect, driving COVID-19 risk
3. Experience of COVID-19 symptoms and having COVID-19 among People with Disabilities	3.1 Rationing of test kits and access to testing for COVID-19 3.2 Experiences with home management of COVID-19 infection 3.3 Navigating unresponsive healthcare

## Theme 1: Awareness and experience of public health measures on COVID-19 among people with disabilities

### Subtheme 1.1: Awareness of COVID-19 and accompanying public health measures among people with disabilities

By November 2022, most people with disabilities knew about COVID-19 broadly as a disease and could explain some of its basic features. This awareness was demonstrated in knowledge about origin and symptoms (including most commonly coughing and sneezing identified by most respondents), as well as transmission modes. Regarding awareness of the methods of preventing COVID-19, a few participants with hearing and speech impairments and most participants with physical, visual and other impairments not affecting communication also knew the methods of preventing COVID-19, under the buzzword of ‘five golden rules’. Additionally, when COVID-19 vaccines were rolled out in Zambia, most respondents with disabilities were aware of COVID-19 vaccines as a prevention measure, being available in the country:

‘Yes, I know about this terrible disease, it has taken a lot of lives around the world.’ (IDI 34, male, 58, physical and mild psychosocial impairment)

‘Through wearing a mask, handwashing or sanitising, coughing in elbow, avoiding crowded places and doing social distance.’ (IDI 09, male, 25, hearing impaired)

‘I heard about corona virus and people [*Ministry of Health Officials*] they were going around asking us if we have already done the ‘inyeleti’ [*taken the COVID-19 jab/injection*].’ (IDI 19, female, 28, epilepsy)

### Subtheme 1.2: Challenges of access to COVID-19 information

#### Challenges with affordability

As mentioned earlier, the majority of participants were living in challenging financial circumstances. Young people with disabilities expressed difficulty in getting funds for internet access because the costs exceeded their ‘pocket money’. They also faced limited opportunities to secure part-time jobs to earn their own income, often relying on male relatives for financial support. Here we found intersections (de Beco 2020) between gender, poverty and disability regarding information accessibility. For instance, a 23-year-old female student participant, who lived in a peri-urban area of a predominantly rural town, with a physical disability indicated:

‘*Ifwe yalikaba*. [Life is hard or things are tough]. Right now, I need bundles to do my assignments, connect with my friends, and learn one or two things about corona. For people with disabilities, it is hard to demand for more pocket money. My sister and I are being kept by an uncle from mum’s side. Nabena kuwaya- wayafye [*he is not a rich man*].’ (IDI 36, female, 23, physical impairment)

The Ministry of Health often disseminated standard text messages with up-to-date information on COVID-19 through phones. Additionally, some people, such as people with vision impairment, required phones with accessibility features, which are often smartphones. However, most participants, particularly those in rural areas, did not own smartphones. A grade 6 boy with vision impairment said:

‘I don’t have a smartphone.’ (IDI 15, male, 15, vision impairment)

#### Information accessibility

**Information accessibility for people with hearing impairments:** Some people with disabilities said COVID-19 health education messaging did not consider the accessibility needs of people with disabilities. This was particularly evident among a few participants with hearing impairments who had low levels of education, no formal sign language instruction and living in a rural area who knew little or nothing at all about COVID-19 as a result. A young man with a hearing impairment who knew sign language also lamented about inaccessible information on COVID-19 among his friends with hearing impairments because of not knowing formal sign language. He suggested a solution for this:

‘Bafunika kupunzhila sign language. Ba usinga sign language ya kumunzhi [*they need to learn formal sign language. They use home signs from the village*].’ (IDI 09, male, 25, hearing impaired)

People with hearing impairment had a history of interactions with health and health promotion services which were largely not respectful of the deaf culture and did not provide sign language interpretation, leading to doubts whether COVID-19 information they received was up-to-date and trustworthy. With so much information and misinformation (infodemic) during the COVID-19 pandemic, participants felt the need to be cautious and expressed the need to have adequate, truthful information on the pandemic, delivered by staff accredited through Organisations of People with Disabilities and professional bodies, who knew both English and Zambian sign language. For instance, a woman who lived in an unplanned settlement with her brother and worked as a volunteer at a local health centre in the city said:

‘I am concerned that the information I have about COVID-19 is not enough. As Deaf people we are sidelined [*When Sign Language Interpretation is not provided*] because messages on the phone, TV updates and social media are the ways in which we get information and we take that as the truth, so you [herself] can worry. The Ministry of Health can get good interpreters from [*name of organisation*] not just anyone to do sign, because the ones I am telling you follow code of ethics [*Sign Language Interpretations’ professional code of ethics*] and are properly trained …’ (IDI 03, female, 49, hearing impairment)

**Information accessibility for people with visual impairments:** Most adult participants with visual impairments said although they could listen to COVID-19 messages on the radio, some stated they would prefer braille. However, the COVID-19 health education fliers distributed were not produced in braille. A few people with visual impairments said technological applications like screen reading software were available on their laptop, and one participant had a smartphone with accessibility features. However, they still experienced accessibility barriers on websites and digital content if these sites were not adapted to be screen reader compatible. To highlight this, a middle-aged male lecturer at a college with visual impairment observed:

‘Many materials in Braille were needed. There is specific software [*text to speech*] like the one I have on my laptop to help navigate. But my experience is that they are not easy to use.’ (IDI 01, male, 52, visual impairment)

**Information accessibility for people with intellectual/cognitive impairments:** Simplified information from the official emergency risk communication committee was aimed to help children understand preventative measures, which could be useful for children and adults with intellectual impairments. Although jingles were included in televised and radio programmes, most parents of children and adults with severe intellectual impairments doubted the ability of the person under their care, to get this information in the generic format. To highlight this, one caregiver of a young man who had a severe intellectual disability felt he was unable to comprehend, a view also reiterated by other parents and caregivers of children and adults with severe intellectual disabilities:

‘No, I do not think that he can understand anything on his own.’ (IDI 18, father, 60, of 25 year old male with multiple impairments)

‘No, she doesn’t talk, and l am not sure she is able to understand, although she hears when people are speaking. She also watches TV. It is ok for me.’ (IDI 07, mother, 53, of 10 year old female with multiple impairments)

Additionally, although they were required to safeguard their children and young adults with severe impairments from getting COVID-19 infection, there were little or no guidelines adapted for the carers in the early part of the COVID-19 pandemic. The absence of adapted guidelines for use was experienced as disempowering, given that COVID-19 was a new disease. Although guidelines were developed later through the association of parents with disabilities, they were not widely circulated to end users:

‘The association [*parents of persons with disabilities*] did not have guidelines at first. We didn’t know what do to. Later, guidelines on what to were developed but not everyone had them.’ (IDI 07, female, 53, of 10 year old female with multiple impairment)

### Subtheme 1.3: Practicalities of following the five golden rules on COVID-19 prevention

#### Negative COVID-19 prevention experience with social and/or physical distancing

**Delays in meeting personal care needs:** Often, people with severe impairments and people with multiple impairments in the study depend on carers for conducting activities of daily living. In many instances, navigating the COVID-19 prevention requirements, particularly for physical distancing, led to strained relationships. People with disabilities said some carers took the golden rules seriously for themselves and had no other information on how to execute care activities safely, leading to delays in receiving personal care. To illustrate this, a young woman with cerebral palsy noted:

‘The challenge with COVID keeping a metre apart was that people with disabilities always needed a helping hand. Like in my case … and at times because some people would want to follow those rules critically before doing anything or touching their relatives, so it was a very big challenge for a person with a disability to get help on time.’ (IDI 22, female, 23, cerebral palsy and physical disability)

**Navigating physically inaccessible public spaces and public transport:** Many participants in urban and peri-urban locations found the built environment a hindrance to keeping physical distancing. While there were visible indicators on where to stand in particular malls, banks and physical marks for maintaining a certain distance in church pews and clinic queues, these were typically not tactile enough for people with visual impairments. Most of our participants with visual impairments do not use walking canes to aid in their independent navigation. Because they require guides and were unable to determine if those around them were adhering to the safety protocols, people with visual impairments were concerned about potential for COVID-19 infection, doubting the extent to which they could rely on others to guide them in public areas and on buses but often had to be helped. To highlight these challenges, two adults with visual impairments said:

‘I do not have a [*walking*] cane … I always need a helping hand because there are stones. When I am away from my family, strangers help me to get on and off the bus. Now this time [*during the pandemic*] people were for the idea of social distancing to prevent the spreading of COVID-19.’ (IDI01, male, 52, visual impairment)

‘We were supposed to sit one meter away, when you want to sit on a bench or even on the bus. But for us blind people this was a challenge because we could not be one meter apart when you need help for [keeping] the same distance.’ (IDI 27, female, 49, visual disability)

#### Barriers to adoption of mask wearing

A few people with visual impairments did not adopt mask wearing, because of physical discomfort or they had difficulty breathing:

‘I have failed to get used to wearing of masks as it makes me very uncomfortable. If there was a way to have them develop those masks which can cover the whole face [*face shield*], I think it would be better because it would leave enough space for air unlike the ordinary ones which block room for air.’ (IDI 01, male, 52, visual impairment)

In some parts of the world, opaque face masks were aimed at making it easier for people who do lip reading to communicate. However, only cloth and medical face masks were often distributed, by both the public and private sectors, during COVID-19 prevention interventions in Zambia. Some participants with hearing impairments who require lip reading to understand what is being communicated could not always wear these cloth and medical face masks. They took risks in public places like schools, hospitals and marketplaces by uncovering their faces. Sometimes, they left their mask hanging below the chin. Most of these participants said they abandoned wearing a mask altogether during a conversation. To highlight this, a male teacher with hearing impairment said:

‘I have no problem wearing a facemask. Every time I go out [*in public spaces*], I wear a mask. But when I am in class with my students, or a fellow deaf person at church or the market, I take it off because first of all they will not see my face. That is how we communicate. we face each other.’ (IDI 23, male, 36, hearing impairment)

#### Handwashing and sanitation

**Inaccessible WASH infrastructure and technology:** During the pandemic, there was an attempt to mount more handwashing facilities in public spaces countrywide. Although most people with disabilities said handwashing was a preferred COVID-19 prevention measure, because of its potential to prevent other diseases as well, only a few said it was an easy measure to follow. Across impairments and geographical location, most participants observed that like pre-pandemic times, the foot pedals were difficult to use and community washing stations were unreachable for wheelchair users, and a view was expressed by a young woman:

‘I was pressed so I called someone to help me. The person guarding the hospital first asked mum to sanitise herself before touching me. After that I had to wash my hands so that there was no barrier when getting help. I couldn’t even reach the wash basin from my wheelchair.’ (IDI 22, female, 23, cerebral palsy and physical disability)

Most participants were not able to wash their hands as often as possible during the COVID-19 pandemic, and owing to cost, some washed their hands with water only.

‘It is just that the cost of soap, hand sanitizer and other alternative to such are costly these days. When there is no soap at home, there is nothing I can do. I wash hands with water that’s all.’ (IDI 33, male, 31, visual impairment)

**Disability and gender roles in household water management:** Prior to the pandemic, climate change issues were said to be causing scarcity of freshwater resources. When the pandemic struck, household members were required to observe frequent hand hygiene, increasing water requirements. However, these demands also placed a huge burden on women and girls because of their domestic roles. For instance, women who resided in rural areas had the responsibility to ensure there was water at home, regardless of their own ability to carry water containers or buckets from water sources like shallow wells and the river. If the water point was near their homes in urban and peri-urban locations, females with hearing and intellectual impairment still had the responsibility of topping up water in containers for members of the household because of erratic supply. To highlight these water challenges, a woman with visual impairment from a rural town in a planned compound with piped water noted:

‘We have a big problem with the water. There is water rationing. We just have water on some days of the week. We draw water in our buckets and store for the period we will not have the running water. For emergencies like cooking beans, we ask from our people [*neighbours*] who have a shallow well.’ (IDI 29, female, 48, visual impairment)

#### Past negative experiences with vaccines

Vaccines were a later addition to the guidelines on COVID-19 prevention. Vaccine acceptability and adoption by study participants varied. Among a few adult participants, who had some level of education, this intervention was a welcome measure. However, a common pattern was not getting much information about the side effects. A man with visual impairment explained his experience:

‘… one thing they did not explain the side effects associated with vaccination. After the vaccine, I was in pain because the site of injection was sore. I felt ill but I did not return to the clinic.’ (IDI 01, male, 52, visual impairment)

Others were reluctant to vaccinate because of past negative experiences, such as attributing disability causation to past vaccination experience and coercion from significant others. For instance, a woman attributed the onset of hip joint problems to a vaccine injection during her childhood observed:

‘I followed all the golden rules except for one, vaccination. I have phobia of injections. When I was young, the [*health worker type*] gave me a vaccine injection on my thigh … From then on, I became unwell, and this led to leg problems, especially around the hip; niyopa nyeleti [*I am afraid of being injected*]. When vaccines came, my sister tricked me. She said there was an event taking place in town and only to find that they were giving jabs [*COVID-19 vaccines*]. I got the first vaccination. The first and second were ok but the last one I got in June was painful.’ (IDI 28, female, 53, physical impairment)

## Theme 2: Experience of othering and stigmatisation of people with disability during the COVID-19 pandemic

### Subtheme 2.1: Disability, COVID-19 stigmatisation

People with disabilities and their families reported that they had already experienced disability-related stigmatisation prior to COVID-19. Contracting COVID-19 or being in a household with someone who had COVID-19 could then lead to additional stigma and exclusion. Some participants reported being shunned by neighbours in these circumstances. One participant was accused of using COVID-19 as a way to ‘get rid of their problem’ namely a child with severe impairments. She said:

‘It [*COVID-19*] has made us think a lot. My son tested positive for corona [*COVID-19*]. Other people in the community were afraid of him but I wasn’t. He is now fine… My greatest worry was that I could get infected with corona [*COVID-19*] and pass it to my girl with impairments. That would have really been hard. People in the village already blame me for ‘contributing to my daughter having a severe a disability’ because of the [*traditional*] belief that I committed incest. She needs my care and now they avoid us, saying with COVID-19 in my house, I have a wicked plan to permanently solve my problem if she dies and they want no hand in it.’ (IDI 07, female, 53, of 10 year old female with multiple impairments)

### Subtheme 2.2: Being labelled as ‘the one spreading COVID-19’

In ordinary times, most adults with disabilities experienced intersecting identities that created spaces of marginalisation and stigmatisation, compared to people without disabilities (Yoshida et al. 2014). Participants said COVID-19 made things worse because of being identified as at higher risk of COVID-19 infection as a person with disability. For instance, a man with post-polio paralysis often needed healthcare for allergies even before the pandemic. He was perceived as higher risk because he went back and forth to health centres. After the pandemic, he experienced severe symptoms of COVID-19 for which he had to seek healthcare at a local clinic, like his allergies. Although he did not test positive for COVID-19, he observed that:

‘*Stigma pa balema tayakapwe* [stigma against people with disabilities will never end], you know very well that people call us names, *balya abekala pacijinga* [the one who sits on a wheeler]. I am also a poor man, as you can see I live in a one room with my second wife and people thought I would not marry gain … now with COVID-19, I fear to sneeze and cough in public because they say you are the ones brining corona [*COVID-19*] because of the way I move to the clinic to get help.’ (IDI 35, male, 56, physical impairment)

Most young people with severe mobility restrictions and all participants with multiple impairments needed physical care from others to function in their daily lives and this involved unavoidable close contact with other human beings. In this study, most of the young people with disabilities had no designated carer and household members took turns to provide such care. A few young people with disability also experienced negative judgements and discriminatory attitudes from other household members because they were seen to be typical of persons likely to spread COVID-19. For instance, a young woman participant said:

‘Before COVID-19 came, people [*other members of the household*] could at least help us move. But after COVID-19, people were afraid to move me from the floor to the bed and from the wheelchair because they thought we [*herself*] could give then COVID-19 because a lot of people come close to us.’ (IDI 20, female, 17, physical impairment)

### Subtheme 2.3: Neglect, driving COVID-19 risk

Participants who became disabled as adults rejected the risk designation based on their personal experience, claiming it put them in a marginalised group deserving of pity. Instead, they emphasised external factors that included government indifference and imposed participation restrictions on people with disabilities, before and during the COVID-19 pandemic. However, they were afraid to voice their concern. As an illustration, a man physically impaired by a traffic accident who worked for an international organisation said:

‘Yes, we were at risk of COVID-19 infection although we were neglected but still, we cannot say that to the government. We only complain in private or when we are with people whom we trust that they cannot report us [*to the authorities*]. Yes, I feel at risk of getting corona [*COVID-19*].’ (IDI 08, male, 53, physical impairment)

## Theme 3: Experience of COVID-19 symptoms and having COVID-19 among people with disabilities

### Subtheme 3.1: Rationing of test kits and access to testing for COVID-19

Among COVID-19 guidelines was instruction to seek healthcare if experiencing symptoms of COVID-19, and testing services were typically provided at health centres around the country. In some instances, younger participants did not access testing services because health centres had few testing resources and were reserving them for severe cases. For instance, a few participants said they were not tested for COVID-19 when they presented to the clinic with symptoms of COVID-19:

‘I drunk warm water and rushed to the clinic when I had strong [*severe*] symptoms of COVID-19. But I was not tested for COVID-19. The [*health worker*] gave me Panadol^©^.’ (IDI 12, female, 21, mild intellectual impairment and physical impairment)

### Subtheme 3.2: Experiences with home management of COVID-19 infection

Besides ensuring their own safety, a few people with disabilities had responsibilities as carers of their own adult children with confirmed COVID-19, placed under home management. Their experiences as contacts of confirmed home management cases of COVID-19 were stressful. As a man from a rural town reflected on his experiences, all members of his household had severe symptoms of COVID, although he said only his daughter tested positive for COVID-19:

‘We were all sick at home and had to be in bedrooms. it was emotionally stressing because you have to see to it that, that person [*daughter*] does not die. I was scared to check on her but with the help of friends, over the phone, they instructed us what to do. This was help because help from the health care workers [*from the hospital*] was not coming as per expectation. We expected a lot of counselling.’ (IDI 08, male, 53, physical impairment)

Apart from the occasional phone call, participants said there was little support for most cases of COVID-19 placed under home management, and they often had to buy medications and use alternative remedies. Most often cited were steaming with eucalyptus leaves, drinking ginger and lemon tea.

### Subtheme 3.3: Navigating unresponsive healthcare

Different influences shaped people with disabilities’ interaction with the health system. These cases highlight gaps between what was also expected and what services they received, given their health needs as people with disabilities. Six out of the 40 participants tested positive for COVID-19. Most of them said it was easy to go to the clinic. For those who required hospitalisation, they reported poor experiences such as lack of prompt attention and inaccessible health services, bringing to the fore how the health system during crisis times provides inappropriate services to persons with disabilities. Participants said services were unresponsive to their treatment needs. Table 3 further highlights these experiences of having to navigate unresponsive healthcare for people with disabilities during the pandemic.

**TABLE 3:** Illustration on navigating unresponsive healthcare.

Case	Description	Comment
Case 1: 27 years old, female, speech impairment	A 27-year-old woman from southern province presented to the general hospital with severe fever following COVID-19 infection. She was treated at the said hospital, and 3 days later, the young lady showed remarked reduction of fever. Given the improvement, she was discharged from the hospital. At the time of the interview – months later – complete resolution of symptoms was noted. She returned to her house in her mother’s compound in a separate small dwelling with her 2-year-old. However, nobody explained to her or her elderly mother why and how she might have gotten COVID-19, what measures to take in future. It remains a puzzle to her.	This case suggests a possible therapeutic success for COVID-19 treatment, but for disability inclusion, this highlights challenges in reasonable accommodations to improve the participation of people with disabilities in their care and achieve good experiences. Part of patient-centred care is explaining probable causes, reassuring clients, and explaining how the medication given to them will work. None of these were explained to her. Treatment without addressing the concerns of this woman may have led to increased likelihood of anxiety.
Case 2: 25 years old, male, intellectual impairments	A 25-year-old male from an urban area was taken to a public hospital. His family felt the public healthcare system was unresponsive to their son’s needs because he was doing poorly but they had to wait 3–4 h ‘just like everyone else’. They attributed this to the limited awareness and the poor judgement of health workers to interpret the seriousness of his condition, hence long hours to receive a diagnosis. After a positive COVID-19 test, his carers switched to private care to ensure he had access to Intensive Care Unit services needed. They incurred a lot of expenses and had to use their savings because the breadwinner’s salary was reduced during the pandemic. He recovered days later and went home.	The case shows how some people with disabilities struggled to get adequate care in an emergency during the pandemic. It is not known whether someone without a disability with similar severity would have had to wait as long as this person. However, deprioritising care for people with disabilities has been commonly reported either because of discrimination or lack of understanding of the seriousness of someone’s symptoms when they cannot communicate themselves. Healthcare workers may need training to better recognise symptoms and their severity in patients who face difficulties communicating (Hashemi et al. 2022).

Note: Please see the full reference list of the article, Seketi, Q.E., Menon, J.A., Michelo, C., Banks, L.M. & Bond, V., 2024, ‘At risk but not adequately included: People with disabilities’ experience of COVID-19 in Zambia’, *African Journal of Disability* 13(0), a1448. https://doi.org/10.4102/ajod.v13i0.1448, for more information.

## Discussion

The experience of people with disabilities was that they were aware of the COVID-19 pandemic as a disease and its associated prevention measures. However, this study also established that people with disabilities faced barriers of access to health information. Using the guidelines themselves presented issues without reasonable accommodations – a missed opportunity to meet their needs. People with disabilities continued to experience othering and stigmatisation during the COVID-19 pandemic and this led to, for instance, being portrayed as the ones spreading COVID-19. The study also found that healthcare needs for COVID-19-related care were not adequately met. Our findings situate how the COVID-19 measures were experienced by people with disabilities. The measures were experienced as barriers in the environment, which often interacted with people’s impairments and health conditions, restricted further their participation in preventative, treatment and care situations, and raised risks of infection and severe disease for people with disabilities. We now discuss these observations, largely focussing on the disabling environments and interaction of contextual factors in these pandemic experiences, which were, largely, exclusion experiences. This discussion is structured around the three themes that arose from the analysis of in-depth interviews of people with disabilities from districts, and the meanings the participants assigned to these experiences.

Firstly, this study found people with disabilities generally had adequate awareness of COVID-19, although many still reported challenges with getting up-to-date information in accessible formats. This is similar to another research in South Africa (Wickenden et al. 2022). Among key gaps *were inadequate adaptions* of health information dissemination for impairment types such as people with vision and hearing impairments, and for people with intellectual impairments. This barrier has been reported in other pandemics such as SARs and Ebola (Kett et al. 2021) and crises such as flooding (Bailie et al. 2022) in which these adaptations were not made. The goal of risk communication is to ensure that people targeted understand risk and adopt behaviours to reduce the threat. To be of value, risk communication, including health information, must be accessible to all people, including people with disabilities (World Bank Group & GFDRR 2017). Duty bearers, especially governments, are responsible for ensuring accessible information, as per the United Nations Convention on the Rights of People with Disabilities’ requirements on accessibility under article 9 (United Nations 2007).

Secondly, people with disabilities were expected to follow largely generic prevention rules (the ‘five golden rules’). These rules were not adapted to individual circumstances such as for people who require caregiving support and could not social distance; opaque masks were not introduced for people who require lip reading and did not factor in poverty contexts and in an unaccommodating environment. For example, some people with disabilities were unable to undertake frequent handwashing because of inaccessible WASH and because soap and other materials were expensive. This finding mirrored other research from before COVID-19 (Wilbur et al. 2021), indicating inaccessible WASH has been a longstanding problem that was exacerbated by the additional sanitation needs during the pandemic.

Using guidelines on COVID-19 was difficult for people with disabilities because the COVID-19 measures required additional resources that were not always within reach. For instance, actual unmet material and financial needs were behind inability to afford or access basics such as electricity, phones, data bundles and facemasks and hand sanitizers. These barriers, around poverty and material deprivation, which negatively impact lived experiences of people with disabilities, were well researched before the COVID-19 pandemic (Trani & Mitchel 2012) and through the Zambia national disability survey, which showed extreme poverty levels among people with disabilities (MCDSS 2018).

Experiences accessing care for COVID-19 symptoms and infection also demonstrated health sector challenges, including long wait times, supply shortages and challenges in meeting the needs of individuals with disabilities. Examples of the latter included lack of training of healthcare workers. Many struggled to get a proper diagnosis because of shortages in test kits, which has been reported also for the general population in LMICs because of inequities in global supply chains (Boro & Stoll 2022). However, for a population that experiences a narrower margin of health (Shakespeare & Officer 2011), studies found the health outcomes of COVID-19 infection are worse compared to people without disabilities (Bosworth et al. 2021) but exact statistics remain largely unknown in countries like Zambia.

This research carries several implications for policy and practice. Government should ensure that multisectoral response measures to future pandemics and other crises are disability inclusive (WHO 2020). Examples of possible interventions include universal designs of WASH infrastructure; targeted communication strategies that provide information in accessible formats, such as braille, large print, or sign language and promotion of the linguistic identity of deaf persons, as per *Persons with Disabilities Act 6 of 2012*; provide adapted guidelines such as ones specifically for caregivers and distribute them widely and on time; and monitor the inclusion of people with disabilities such as by including disability indicators in the Ministry of Health’s District Health Information System 2 (DHIS2) tool. Further improving systems so that they are more inclusive of people with disabilities can both reduce the impact of future pandemics on people with disabilities and contribute to improving systems for people with disabilities in the longer term. For example, healthcare facilities should be equipped to accommodate the needs of people with disabilities, including training staff, providing accessible testing and treatment options and sign language interpreters. Within the idea of ‘nothing about us without us’ for example, Organisations of People with Disabilities (OPDs) should be involved in all these measures, both in designing them and in their implementation (McBride-Henry et al. 2023; WHO 2021), although additional resources may be needed to strengthen these organisations and expand their constituencies. These accessible measures could go a long way in contributing to the promotion and protection of the right to health during emergencies.

Further research is needed. Through the National Health Research Institute and ethics bodies, researchers should be encouraged to collect data, which is inclusive of disability indicators. For instance, in quantitative research, researchers should use appropriate tools such as the Washington group short set of questions in surveys because this inclusion enables analysis of disability as a predictor of health outcomes, and/or measures effects of other phenomena on disability. This is needed to address other gaps in disability-specific data, required to provide more evidence on health inequities, thereby contributing to reduction of risks of infection during pandemics and leave no one behind. In qualitative research, there is need to also explore disability inclusion in policies and strategies, to improve health outcomes for people with disabilities in LMICs like Zambia during crisis times. Additionally, further research is needed on measures that address social determinants of health such as chronic material poverty and deprivation, stigma and intersectionality. Lastly, researchers should engage in implementation research to learn more about barrier removal and conduct research on feasibility of disability-inclusive measures such as harmonising the sign language needs and cultural needs for people with hearing impairments. Implementation research should also study adoption, reach and effectiveness of interventions meant to improve access to health services for different impairment groups and marginalised populations during pandemics and other public health crises.

### Strengths and limitations

This study has several strengths like the recruitment of a diverse pool of participants across geographical locations, impairment types and gender. Still, there are some limitations to consider. To begin with, our research was conducted in only 11 of 116 districts in Zambia. Most participants were recruited purposively through OPDs – often those affiliated with OPDs are very engaged in the disability community and may have received support during COVID-19 from these organisations. We did include some participants through snowball sampling who were not part of the OPDs, which may have minimised this issue.

## Conclusion

In conclusion, we note that in this population, COVID-19 interventions were largely unresponsive to the needs of people with disabilities, exacerbating the risk of exposure to infection, stigma and discrimination and other disadvantages like unmet triple health needs. The lack of adaptations to deal with the increased risk of COVID-19 infection and serious illness for people with disabilities indicates a need for disability inclusion using both targeted and mainstreaming strategies (twin-track approach) in multisectoral response during pandemics. In future pandemic control, interventions should be based on the lived reality of people with disabilities and adapted to their requirements. Hence, most of the response and prevention methods should have appropriate adaptations like emergency risk communication in braille, audio formats, sign language interpretation to ensure their usability, and target barriers of access to services could potentially contribute to a more disability-inclusive response. Further studies are needed to quantify the gaps in access to health and to explore policies and strategies to improve health outcomes for people with disabilities in LMICs like Zambia, thereby contributing to the reduction of health inequities during crises and promoting the achievement of the larger sustainable development goals.
